# The Giant Magnet in the Extraction of Foreign Bodies from the Eye

**Published:** 1913-02

**Authors:** 


The Giant Magnet in the Extraction of Foreign Bodies from the Eye.—Willis O. Nance, Chicago (*Journal Oph. and Oto.-Laryng.,* October, 1912), emphasizes the point that cases of injury in which the coats of the eye are ruptured demand the care of the skilled specialist. In perforation of the cornea the flying missile very rarely lodges in the anterior chamber but almost invariably enters deeper into the eye before coming to rest. It is in these cases that the giant magnet is of the greatest value. The importance of proper technic in the removal of foreign bodies from the vitreous is second only to the operation for cataract in surgical ophthalmology. Unskilled maneuvers may easily make a bad matter worse. The shape and size of the foreign body is of great importance. If the locataion is shown by the X ray to be in a convenient position it may be better to remove it by a new opening through the sclera. A few cases have been reported where foreign bodies have been removed after negative X-ray report. Early removal is essential. A large proportion of cases of sympathetic inflammation occur in eyes in which a foreign body exists in the other eye. The giant magnet is an instrument of untold value. Without it many eyes would go to destruction and many more cases of sympathetic uveitis would occur.

*Note.*—In each of the eye dispensaries in Buffalo there is a giant magnet, supplied by Erie county, and, for physicians, accessible at all times without any charge.

